# Effect of amide protoporphyrin derivatives on immune response in *Apis mellifera*

**DOI:** 10.1038/s41598-022-18534-9

**Published:** 2022-08-24

**Authors:** Mariusz Trytek, Katarzyna Buczek, Agnieszka Zdybicka-Barabas, Iwona Wojda, Grzegorz Borsuk, Małgorzata Cytryńska, Agnieszka Lipke, Dorota Gryko

**Affiliations:** 1 Department of Industrial and Environmental Microbiology, Institute of Biological Sciences, Faculty of Biology and Biotechnology, Maria Curie-Skłodowska University, Akademicka 19, 20-033 Lublin, Poland; 2 Department of Immunobiology, Institute of Biological Sciences, Faculty of Biology and Biotechnology, Maria Curie-Skłodowska University, Akademicka 19, 20-033 Lublin, Poland; 3 Institute of Biological Basis of Animal Production, Faculty of Biology, Animal Sciences and Bioeconomy, University of Life Sciences in Lublin, Akademicka 13, 20-950 Lublin, Poland; 4 Department of Inorganic Chemistry, Institute of Chemical Sciences, Faculty of Chemistry, Maria Curie-Skłodowska University, M. Curie Skłodowska Sq. 2, 20-031 Lublin, Poland; 5 Institute of Organic Chemistry, Polish Academy of Sciences, Kasprzaka 44/52, 01-224 Warsaw, Poland

**Keywords:** Entomology, Parasitic infection, Reverse transcription polymerase chain reaction, Fluorescence imaging, Parasite host response, Antimicrobial responses, Enzymes, Antifungal agents

## Abstract

The intracellular microsporidian parasite *Nosema ceranae* is known to compromise bee health by induction of energetic stress and downregulation of the immune system. Porphyrins are candidate therapeutic agents for controlling *Nosema* infection without adverse effects on honeybees. In the present work, the impact of two protoporphyrin IX derivatives, i.e. PP[Asp]_2_ and PP[Lys]_2_, on *Apis mellifera* humoral immune response has been investigated in laboratory conditions in non-infected and *N. ceranae-*infected honeybees. Fluorescence spectroscopy analysis of hemolymph showed for the first time that porphyrin molecules penetrate into the hemocoel of honeybees. Phenoloxidase (PO) activity and the expression of genes encoding antimicrobial peptides (AMPs: abaecin, defensin, and hymenoptaecin) were assessed. Porphyrins significantly increased the phenoloxidase activity in healthy honeybees but did not increase the expression of AMP genes. Compared with the control bees, the hemolymph of non-infected bees treated with porphyrins had an 11.3- and 6.1-fold higher level of PO activity after the 24- and 48-h porphyrin administration, respectively. Notably, there was a significant inverse correlation between the PO activity and the AMP gene expression level (r =  − 0.61696, *p* = 0.0143). The PO activity profile in the infected bees was completely opposite to that in the healthy bees (r =  − 0.5118, *p* = 0.000), which was related to the changing load of *N. ceranae* spores in the porphyrin treated-bees. On day 12 post-infection, the spore loads in the infected porphyrin-fed individuals significantly decreased by 74%, compared with the control bees. Our findings show involvement of the honeybee immune system in the porphyrin-based control of *Nosema* infection. This allows the infected bees to improve their lifespan considerably by choosing an optimal PO activity/AMP expression variant to cope with the varying level of *N. ceranae* infection.

## Introduction

Research conducted in the past decade has provided numerous insights into honeybee immunity. In response to the presence of pathogens, honeybees activate many molecular pathways and various defense mechanisms engaging both cellular and humoral innate immune response^1–3^. The humoral response includes e.g. activation of the pro-phenoloxidase (proPO) system and induction of the synthesis of antimicrobial peptides^4^. The activation of the proPO system plays an important role in the insect humoral immunity^5,6^, which leads to the synthesis of melanin, i.e. a dark pigment^7^. Melanization is a key immune mechanism used by arthropods to support cellular immunity^8,9^ and is induced faster than immune responses that depend on changes in gene expression^10^. The induced humoral immunity additionally involves such antimicrobial peptides (AMPs) as abaecin, apidaecin, defensin, and hymenoptaecin to control the invasion of a broad spectrum of pathogens, including *Nosema* and *Paenibacillus larvae*^2,11,12^.

*Nosema ceranae* and *Nosema apis* microsporidia are pervasive and widespread honeybee pathogens associated with colony declines^13–15^. They are obligate intracellular parasites infecting midgut epithelial cells of host larvae and adult honeybees^16,17^. Currently, both microsporidian species have a worldwide distribution as causative agents of nosemosis in western honeybees^18,19^. However, *N. ceranae* shows higher virulence, and infections caused by this species have become more common than *N. apis* infestations^20,21^. Compared with other honeybee species, *Apis mellifera* is especially susceptible to *N. ceranae* proliferation. It is suggested that the lower immune response might be one of the factors contributing to the high prevalence of these pathogens in *A. mellifera*^3,22^. The regulation of immune functions can be associated with the insects responding to clear the infection and with the infecting *N. ceranae* altering host gene expression to promote its survival^23^.

*N. ceranae* is considered to cause major health problems associated with morbid physiological impairments leading to reduction in bee lifespan^20,24,25^. *N. ceranae* infection causes energetic stress and compromises bee health by affecting the immune system, including downregulation of immunoregulatory genes and disturbances in the host amino acid metabolism^1,23,26^. Disturbances in protein metabolism may be one of the strategies adapted by *N. ceranae* to suppress the immune response in bees^26^ and promote its survival. As demonstrated in previous studies, *N. ceranae* infection downregulates AMP genes and some other immune-related genes, including glucose dehydrogenase (GLD) and vitellogenin (Vg)^11,23,26,27^.

Regardless of the mechanism of the immune response of honeybees to pathogens, studies are focused on the link between protein nutrition and immunity^28^, the role of honeybee core microbiota in immune defense^29–31^, and the effect of pesticides (e.g. insecticides) on the immune system^30,32–34^. Much less attention has been paid to investigations of the interaction of the honeybee immune system with various compounds used in the treatment of fungal and bacterial diseases in honeybees^12,35^. Organic extracts and natural supplements have been suggested to be immunomodulatory agents in the treatment of *Nosema* infection; however, no in-depth studies have been conducted to prove their immunological activity^12,36–40^.

Porphyrins are good candidates to be photosensitizers in photodynamic therapy^41^. Recently, porphyrins have been postulated to be used for controlling *N. ceranae* infection without light irradiation and with no adverse effect on honeybees^42,43^. It has been suggested that protoporphyrin IX derivatives conjugated with aspartate and lysine moieties can act directly against microsporidia, which was associated with their active transport into the spore and disruption of their cell wall^44^.

**Figure 1:**
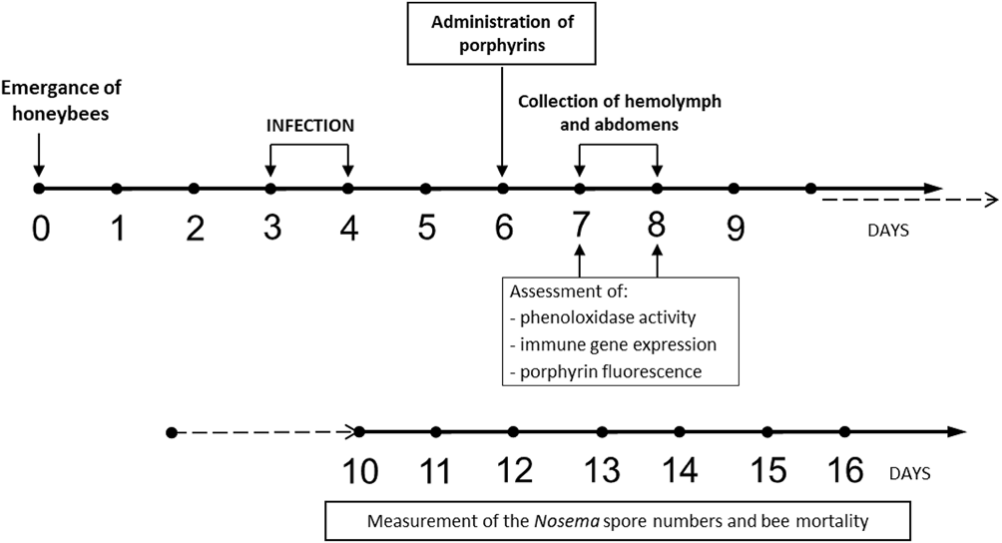
Flowchart of the experiments analyzing the effect of protoporphyrin IX derivatives on *Apis mellifera* humoral immune response, number of *Nosema ceranae* spores in bees, and bee survival.

The aim of the present study was to determine the effect of two different amide derivatives of protoporphyrin IX, i.e. PP(Asp)_2_, and PP(Lys)_2_, on the *Apis mellifera* immune system, which can contribute to the porphyrin-based control of infection with *Nosema* microsporidia and improve bee survival. The level of phenoloxidase activity in the hemolymph and immune-related gene expression was determined after porphyrin treatment in both healthy and *N. ceranae*-infected honeybees (Fig. 1).

## Results

### Level of *Nosema ceranae* infection and survivability of honeybees

**Figure 2:**
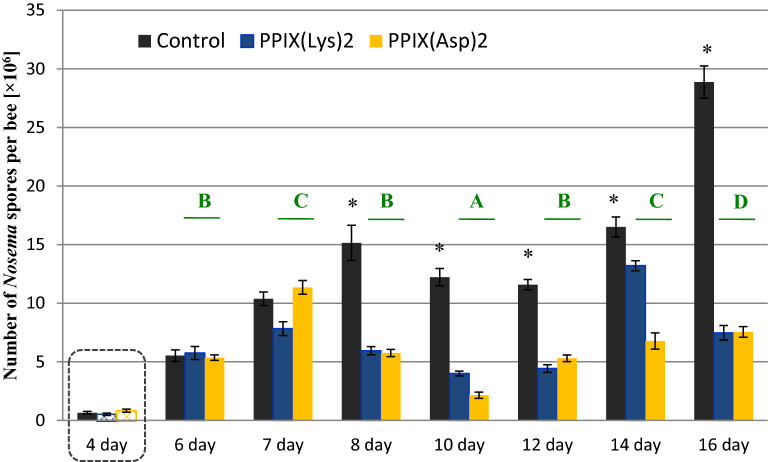
Effect of amide derivatives of protoporphyrin IX on the number of *Nosema ceranae* spores in infected *Apis mellifera*. Statistically significant differences between the infected control group and porphyrin-treated groups on a particular day are indicated with asterisks; block letters (A,B,C,D,E) indicate statistically significant differences between the days in the porphyrin groups. The dashed line indicates groups of infected bees prior to porphyrin administration. The error bars represent standard errors of data.

The number of *N. ceranae* spores on the second day of the infection was in the range of 0.46–0.48 × 10^6^ spores per bee (day 4, Fig. 2), and the differences between the groups were not significant. Similar spore numbers are known to cause an infection in worker bees^24,45^. Two days post inoculation (p.i.) (day 6, Fig. 2), the spore loads in honeybees in all experimental groups increased to 5.5–6.1 × 10^6^ spores per bee and were not significantly different from one another (F_(2, 57)_ = 1.16; *p* = 0.393), which means that the infection procedure was successful and the honeybee groups were evenly inoculated. The differences in the spore loads between the groups were observed after day 3 p.i. (after day 7 of the experiment) (F_(2,57)_ = 89.23; *p* < 0.001). On day 16, the level of infection in the control infected bees increased to 28.9 × 10^6^ spores per bee, whereas the level of infection in the PP(Asp)_2_-treated and PP(Lys)_2_-treated groups gradually decreased up to day 12, and changed statistically significantly after the next four days (*p* = 0.043).

**Figure 3:**
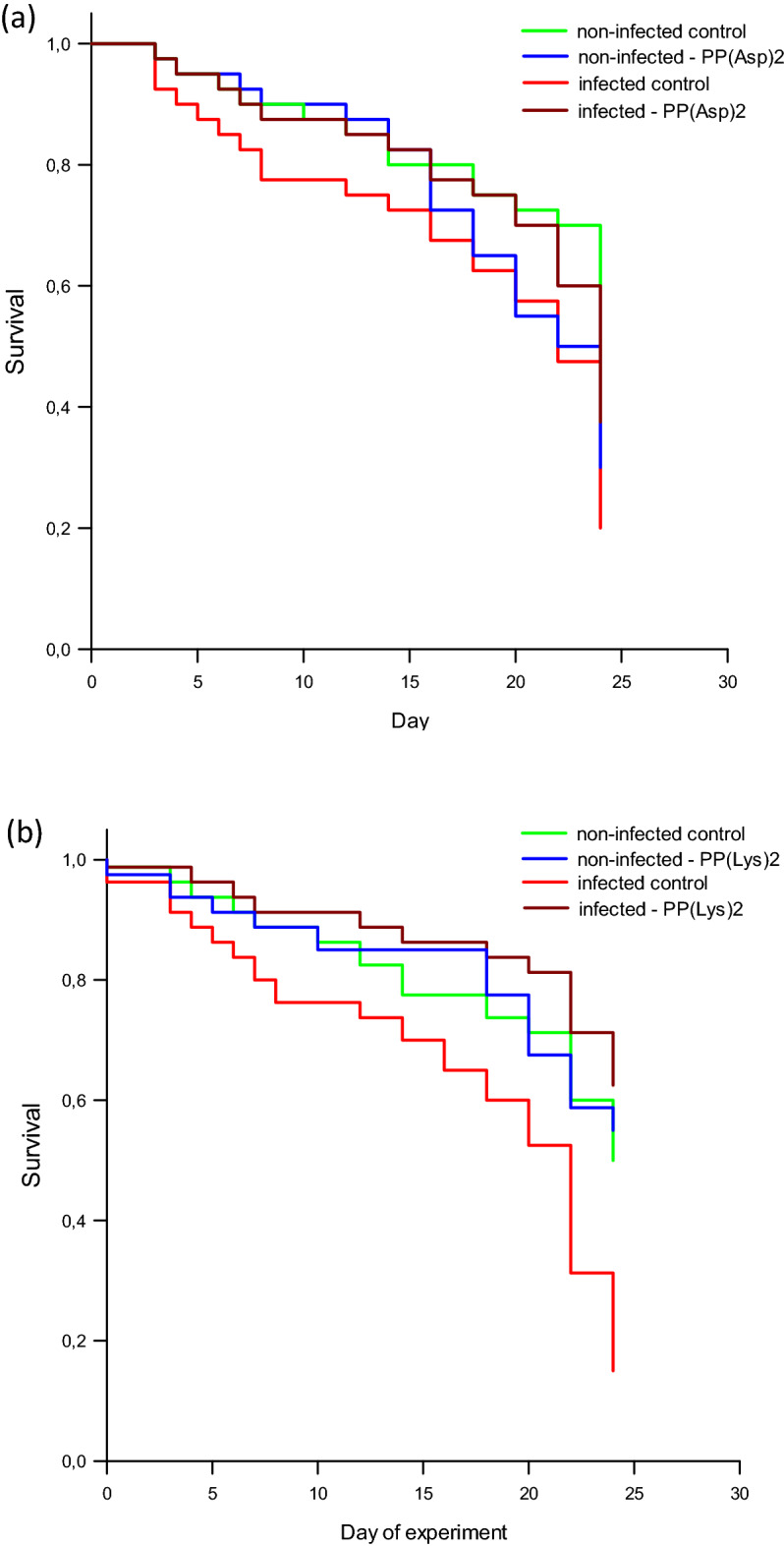
Kaplan–Meier survival curves for uninfected and *N. ceranae-*infected honeybees treated with protoporphyrin IX derivatives (**a**) PP(Asp)_2_ and (**b**) PP(Lys)_2_ administered with the sucrose syrup supplement.

The survival analysis showed that the *Nosema* infection led to a significant increase (*F* = 9.29; *p* = 0.00917) in the mortality of the honeybees, compared with the non-infected control honeybees (Fig. 3). Both PP(Asp)_2_ and PP(Lys)_2_ had no significant impact on the survival of healthy honeybees during the entire experiment (*p* = 0.07). The mortalities of the infected honeybees exposed to PP(Asp)_2_ and PP(Lys)_2_ were significantly lower than in the control infected honeybees (*F* = 18.27; *p* = 0.0412 and *p* = 0.0001, respectively), which reached the highest mortality rate (~ 85%) at the end of the experiment (Fig. 3a,b).

### Determination of the presence of porphyrin in honeybee intestines and hemolymph

**Figure 4:**
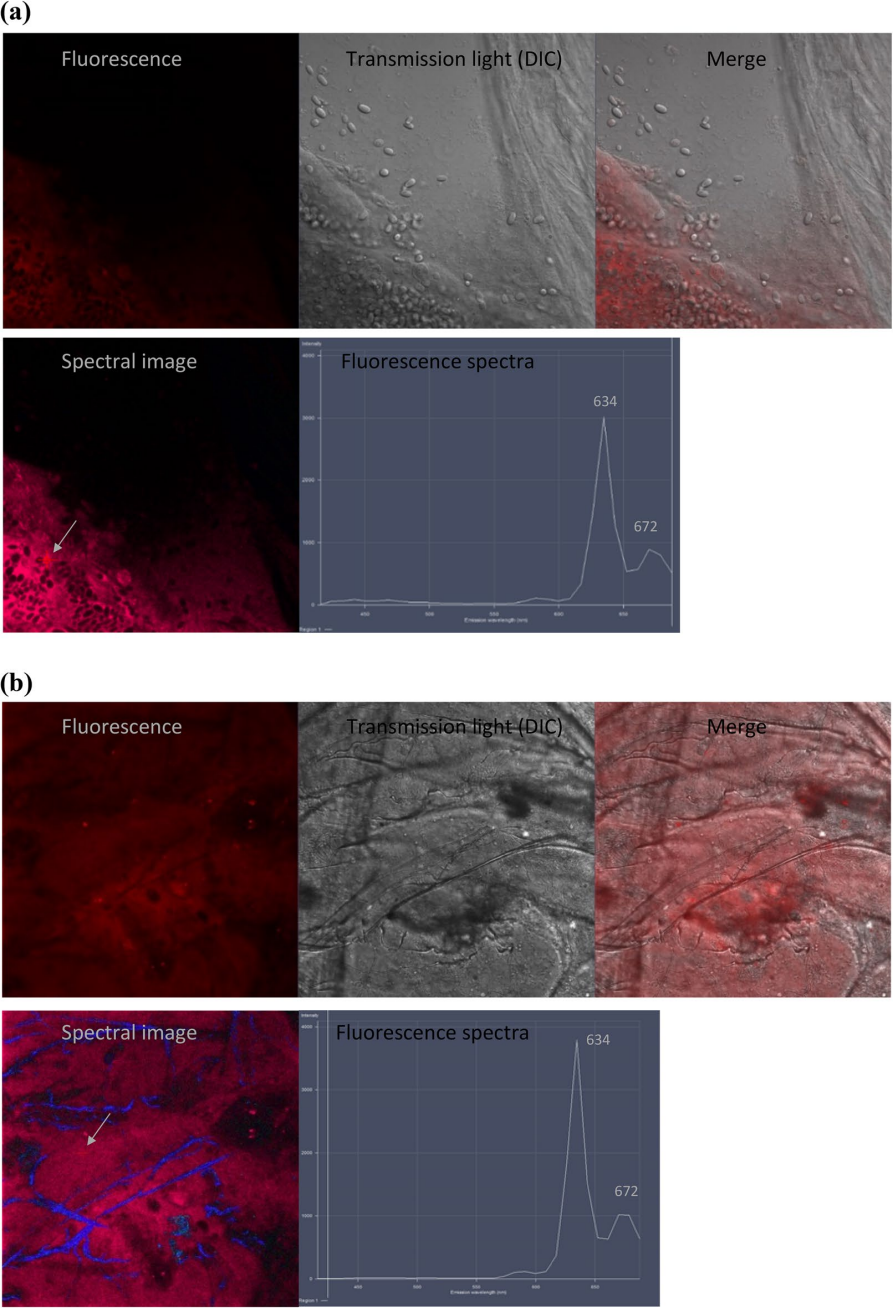
Confocal microscopic images of the midgut segment with porphyrin accumulation in the area of epithelial cells: (**a**) with anchored *Nosema ceranae* spores in infected honeybees and (**b**) free of *N. ceranae* spores in non-infected honeybees (λ_exc_ = 405 nm). DIC—differential interference contrast; corresponding fluorescence spectra were collected from the points indicated by arrows.

To verify the hypothesis that PPIX derivatives can induce immune response in bees to combat *Nosema* pathogens, we first analyzed the potential transfer of porphyrins from the midgut to the hemolymph of the honeybees. Porphyrin molecules were detected in the area of midgut epithelial cells by porphyrin fluorescence measurements in both the infected and non-infected honeybees treated with porphyrins. The segments of the midgut containing accumulated porphyrins emitted red fluorescence with emission spectra characteristic of porphyrins with two maxima at 634 nm and 672 nm (Fig. 4a,b; spectral image panel). Moreover, a tendency of the porphyrins to accumulate near anchored *Nosema* spores was observed.

**Figure 5:**
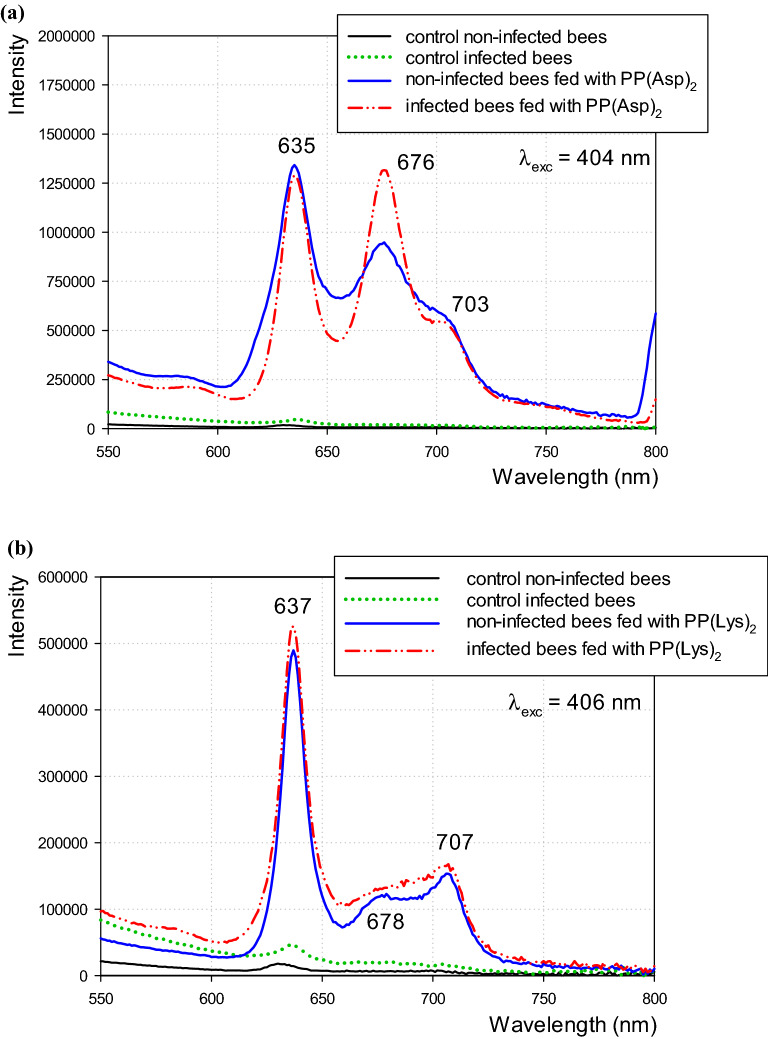
Fluorescence spectra of the honeybee hemolymph showing the presence of protoporphyrin IX amide derivatives (**a**) PP(Asp)_2_ and (**b**) PP(Lys)_2_ inside the hemocoel.

The emission spectra of the hemolymph of both healthy and *Nosema*-infected honeybees treated with porphyrins (Fig. 5a,b) contained three bands with maxima at 635, 676, and 703 nm for PP(Asp)_2_ and at 637, 678, and 706 nm for PP(Lys)_2_. In the case of PP(Asp)_2_, a more intense band at 676 nm was observed in the spectra of hemolymph from the infected honeybees than in the spectra of the healthy honeybees. The fluorescence intensities of the PP(Lys)_2_-containing hemolymph did not differ significantly between the experimental groups. No fluorescence signals characteristic of porphyrins were observed in the hemolymph collected from honeybees fed with pure sugar syrup. Only a small fluorescence band was detected near 635 nm in the group of the porphyrin-untreated *Nosema*-infected honeybees. The fluorescence spectra of PP[Asp]_2_ and PP[Lys]_2_ in water, each with two well-resolved emission peaks (in the region of 600–650 nm and 650–720 nm), are presented in Supplementary Fig. S2.

### Effect of porphyrins on phenoloxidase activity in honeybees

**Figure 6:**
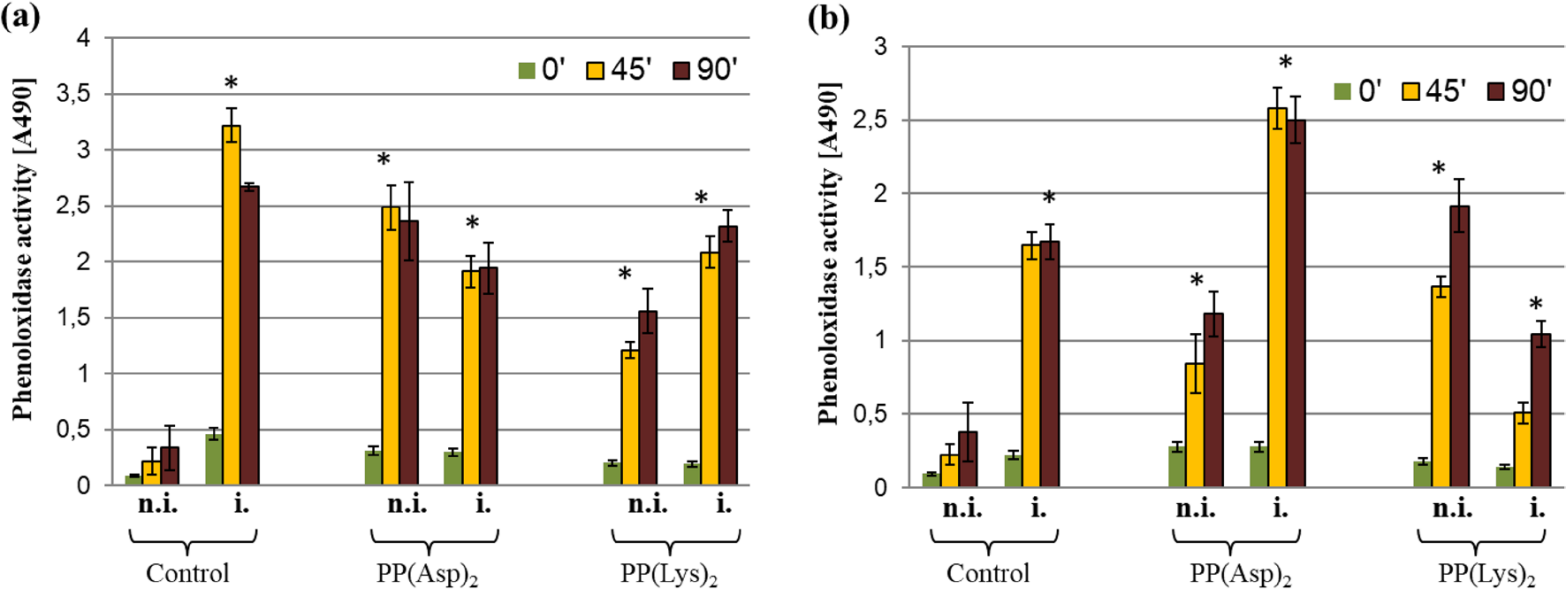
Phenoloxidase activity in honeybees on days 3 (**a**) and 4 (**b**) post infection; n.i.—non-infected honeybees; i.—*Nosema*-infected honeybees; Controls—honeybees fed with pure sucrose syrup; PP(Asp)_2_ and PP(Lys)_2_—honeybees fed with sucrose syrup containing porphyrin.

To check whether porphyrins passing from the digestive system to the hemolymph of the honeybees affected their humoral immune response, the level of phenoloxidase (PO) activity in the hemolymph was investigated. The highest level of PO was recorded on day 3 post infection in the porphyrin-untreated *Nosema*-infected honeybees (Fig. 6a). After 4 days post infection (Fig. 6b), the bees in this group showed significantly lower PO activity (*p* = 0.036). The non-infected control bees had the lowest PO activity among all the investigated groups. It was approximately 14.5- and 7.3-fold lower on days 3 and 4 post infection, respectively, compared with the *Nosema*-infected honeybees (*p* = 0.0001 and *p* = 0.007, respectively). The level of PO activity in the hemolymph of the non-infected bees receiving porphyrins was 11.3-fold [PP(Asp)_2_] and 5.5-fold [PP(Lys)_2_] higher after 24 h of administration of the porphyrins than in the non-infected control group (*p* < 0.001). The honeybees treated with PP(Asp)_2_ and PP(Lys)_2_ for 48 h had 3.8-fold and 6.1-fold higher PO activity, respectively, than that observed in the non-infected control bees (*p* < 0.01). In turn, slightly lower PO activity in the hemolymph of the porphyrin-treated *Nosema*-infected bees was noted 24 h after administration of the porphyrins, compared with the hemolymph of the infected control bees (*p* < 0.05). Significant differences in PO activity between the *Nosema*-infected groups were observed after 48 h of administration of the porphyrins (F_(2, 42)_ = 93.04; *p* < 0.001). In comparison with the infected control honeybees, the level of PO in the hemolymph was 1.5-fold higher for PP(Asp)_2_ but substantially (3.2-fold) lower in the case of PP(Lys)_2_.

### Immune-related gene expression

**Figure 7:**
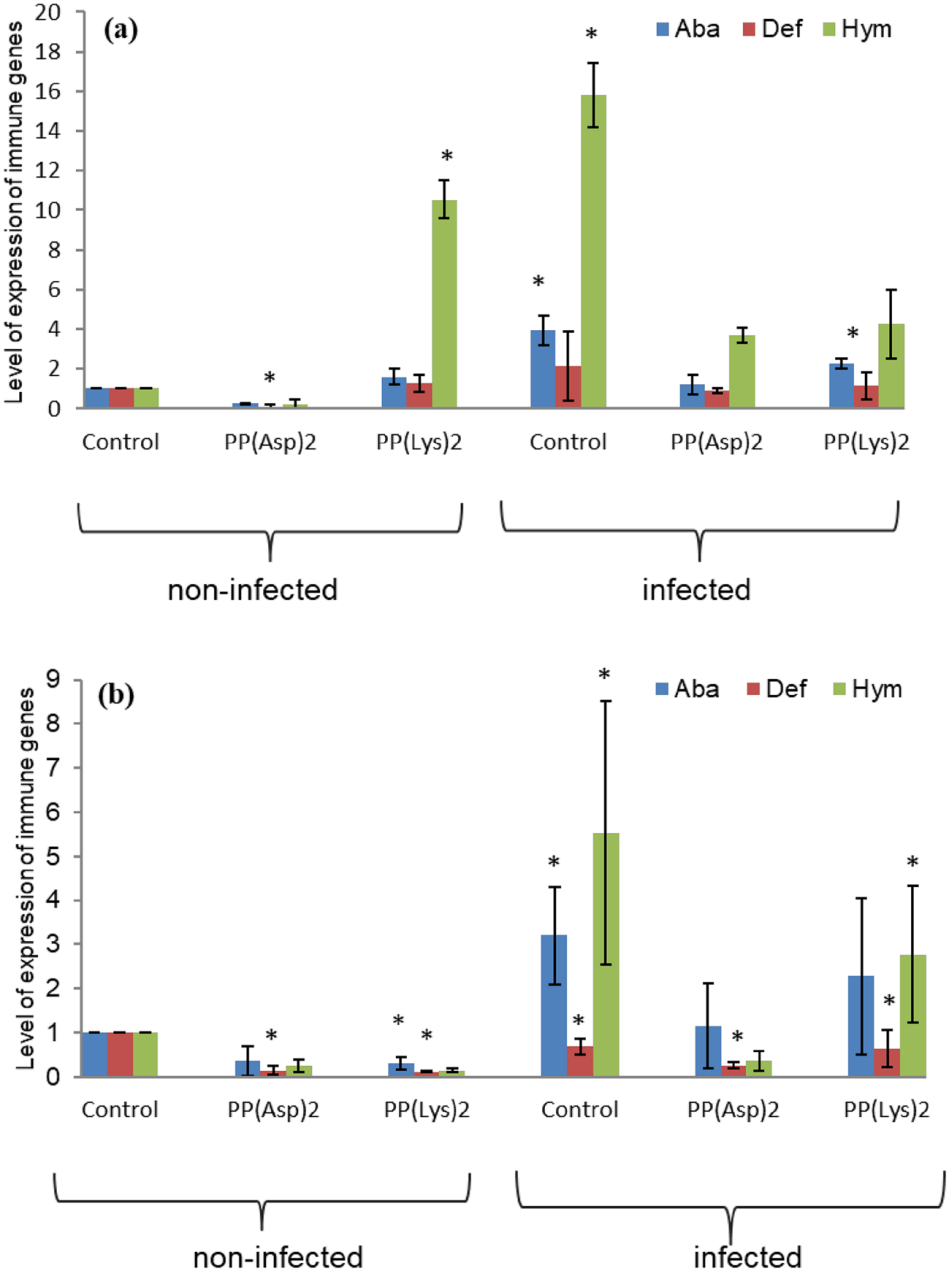
Effect of porphyrins PP(Asp)_2_ and PP(Lys)_2_ on immune-related AMP gene expression in honeybees (**a**) on day 3 post *N. ceranae*-infection; (**b**) on day 4 post *N. ceranae*-infection. Aba—abaecin, Def—defensin, Hym—hymenoptaecin. Non-infected control and infected control bees fed with sucrose syrup only. Bars marked with asterisks are statistically significantly different from the non-infected control; error bars represent standard errors of data.

The highest level of the expression of immune genes was observed on day 3 post infection in the porphyrin-untreated *Nosema-*infected bees (Fig. 7a; Supplementary Table S3). On day 4 post-infection (Fig. 7b; Supplementary Table S4), the bees in this group showed slightly lower expression of Aba and approximately threefold lower activity of the Def and Hym genes than those on day 3 p.i.. Compared with the non-infected control bees, lower transcript levels of all three immune genes were observed in the non-infected bees treated with porphyrin PP(Asp)_2_ both 24 and 48 h after porphyrin administration (on days 3 and 4 p.i., respectively). However, a statistically significant difference was observed only for Def [H_(2, N=18)_ = 12.23, *p* < 0.005], and subtle differences between the treatments were also apparent for Aba (*p* > 0.05). The non-infected bees treated with porphyrin PP(Lys)_2_ showed slightly higher expression of immune genes, compared with the non-infected control after 24 h of porphyrin administration, with statistically significant difference observed for Hym [H_(2, N=18)_ = 15.76, *p* < 0.001]. After 48 h of PP(Lys)_2_ administration, the gene expression decreased relative to the non-infected control [*p* < 0.05 (Aba), *p* < 0.005 (Def)]. The decrease was not statistically significant in the case of Hym. The *Nosema*-infected bees treated for 24 h with PP(Asp)_2_ showed lower expression of immune genes relative to the infected control, and a statistically significant difference was observed only for Aba [H_(2, N=18)_ = 15.21, *p* < 0.001]. After 48 h of PP(Asp)_2_ treatment, significantly lower expression of all immune genes was noted for Aba [F(2, 15) = 4.35, *p* < 0.05], Def [F(2, 15) = 8.65, *p* < 0.01], and Hym [F(2, 15) = 28.68, *p* < 0.001], relative to the infected control insects. The infected honeybees treated with PP(Lys)_2_ showed a slight decrease in the transcript levels of immune genes after both 24 and 48 h of treatment. A statistically significant difference was observed only for the Aba gene expression after 24 h of the porphyrin administration, compared with the infected control (*p* < 0.05).

The groups of infected honeybees treated with the porphyrins showed very similar levels of expression of the Def and Hym genes after 24 h of the treatment (day 3 p.i.). In turn, they decreased to varying degrees for PP(Asp)_2_ and PP(Lys)_2_ after 48 h of the treatment (day 4 p.i.). The expression of the Def and Hym genes was threefold and 7.8-fold lower in the group of the PP(Asp)_2_-treated bees than in the PP(Lys)_2_-treated bees, respectively (Fig. 7; Supplementary Table S3).

## Discussion

Development of strategies that can efficiently stimulate the honeybee immune system with no simultaneous threat posed to insects, humans, and the environment is of great importance. Porphyrins have been documented to act directly on *N. ceranae* microsporidia; however, their potential effects on honeybee immunity have yet to be explored. Before analyzing the impact of porphyrins on the immune system in both the non-infected and *Nosema-*infected honeybees, we verified that the parasite proliferated in the host and significantly reduced its lifespan, as observed in previous studies^1,3,37,46,47^. The present work confirmed the negative impact of *N. ceranae* on the bee lifespan. Significant differences in mortality between the non-infected and infected honeybees were observed from day 3 post infection (p.i.) (*p* < 0.009) (Fig. 3), which was related to the significant increase (*p* < 0.001) in spore loads in the infected honeybees on day 2 p.i. (day 6 compared with day 4) reaching 3 × 10^7^ spores per bee at the end of the experiment (Fig. 2).

Microsporidian infection can alter both local and systemic innate immune responses in the insects^48,49^. Several immune components in honeybees have been found to be regulated on days 3 and 6 p.i. with a *Nosema* infection level below 0.5 × 10^5^^11^ and at 5 × 10^5^ spores per bee^23^, respectively. Other data demonstrated that immune factors were induced by a *Nosema* load of approximately 10^5^^3^ and an infective dose of 0.2 × 10^6^ spores per bee^12^ on days 4 and 6 p.i., respectively.

Since insects can mount a constitutive (phenoloxidase, PO) and induced (antimicrobial peptides, AMPs) immune response to diverse pathogens^2,6,50^, we determined PO activity and the expression of three immune-related genes encoding known AMPs (Aba, Def, and Hym) in non-infected and *N. ceranae*-infected honeybees.

As shown in the present study, at the level of infection of 1.1 × 10^7^ spores per bee on day 3 p.i., the honeybees showed substantially higher PO activity in the hemolymph than the non-infected control bees. However (Fig. 6a), on the next day, the PO activity substantially decreased in the *Nosema*-infected bees concurrently with the increase in the spore load (Fig. 6b). These results are consistent with those reported by Sinpoo et al.^3^, who showed significant upregulation of the PO gene 4 days after *N. ceranae* inoculation, followed by a decrease in its expression 7 days after infection. Antúnez et al.^26^ have also shown that the level of PO gene expression increased (though not significantly) during the first four days post infection to be subsequently reduced on day 7 p.i., compared with control bees. Significant reduction of PO enzyme activity was found in *N. ceranae* infected-honeybees after day 18 p.i.^51^. Similarly, our study of the defense gene expression showed a decrease in the transcript levels of the AMP genes in the infected honeybees on day 4 p.i., which was preceded by substantial induction thereof on day 3 p.i. relative to the healthy bees (Fig. 7; Supplementary Table S3). The present work confirms that *A. mellifera* use a common defense mechanism against microsporidian infection^3,24^ and suggests that, following the initial induction, the immune system of honeybees is downregulated by *N. ceranae* over time. This corresponds to the postulate that *N. ceranae* suppresses humoral and cellular defense mechanisms in honeybees^11,23,26^. The partial immune suppression induced by the *Nosema* infection of the honeybees may have begun around day 4 p.i., as evidenced by the reduced lifespan of the infected bees (Fig. 3).

However, the immune system of the *N. ceranae*-infected honeybees did not seem to be completely suppressed within 4 days of the infection, since both the PO activity and the AMP gene expression persisted at a higher level in the infected honeybees than in the healthy insects (with the exception of Def showing a similar gene transcript level). An earlier study has reported significant reduction of Aba, Hym, GLD, and Vg expression in honeybees seven days after infection^26^. In contrast, other investigations indicated that the transcripts of antimicrobial peptide genes were suppressed in workers already on days 3 and 6 p.i. with *N. ceranae*^11^ or strongly downregulated on day 5 p.i., compared with non-infected controls, but not on days 10 and 15 p.i., except for the hymenoptaecin gene, which was upregulated on day 15 p.i.^23^. The time-dependent inconsistence in immune-related gene expression in *Nosema*-infected bees has already been recorded by Antúnez et al.^26^, Chaimanee et al.^11^, and Sinpoo et al.^3^. The variation in the gene expression patterns is most likely related to some differences between experimental designs and genetic differences between honeybees. Moreover, the infection status (spore load), age polyethism, and ageing of bees may exert different effects on the expression of immunity genes, which is dynamic over time^23^.

In our further study, to connect the immune changes induced by the *N. ceranae* infection to porphyrins, the impact of porphyrins on humoral immune response was investigated in the *Nosema*-free and *Nosema*-infected bees in conjunction with the infection level and bee survival. As expected, both porphyrins administered to the honeybees at the 100 μM concentration decreased the spore loads significantly (by 74%) in the infected individuals after 16 days of the experiment. As we showed in the previous work^44^, the destruction of microsporidia by porphyrins is associated with their active transport into live spore cells. Thus, part of the PP(Lys)_2_ or PP(Asp)_2_ molecules consumed by honeybees penetrate and destroy the spores; however, the further action of the other molecules in the bee body has been undefined so far. Interestingly, the excess porphyrin molecules were revealed in the present study to have a tendency to accumulate in the area of midgut epithelial cells (Fig. 4). Therefore, in the next step of the study, we analyzed whether the porphyrin molecules penetrate into the hemolymph and thus activate the humoral response of the immune system. The spectrofluorimetry technique facilitated identification of porphyrins in the bee hemolymph. The hemolymph of both the non-infected and infected honeybees exposed to porphyrins evidently showed red fluorescence with two main emission bands characteristic of the corresponding porphyrins (Fig. 5, see Supplementary Fig. S2). This proved their transfer from the midgut to the hemocoel of honeybees. The red shift in the bands (from 622–624 to 635–637 nm) and the split of the bands in the 650–720 nm region into two peaks in the emission spectra of porphyrins in hemolymph in relation to their spectra in water (Supplementary Fig. S2) suggest either an interaction of porphyrins with hemolymph components or modification of the porphyrin structure in the environment of the bee midgut and/or hemolymph. Notably, the comparison of the hemolymph spectra of the infected and non-infected honeybees treated with PP(Asp)_2_ revealed a distinct change in the fluorescence intensity of the 635 nm band versus the 676 nm band. This may indicate that the hemolymph of *Nosema*-infected bees may contain factors that interfere with the porphyrins.

It was clearly demonstrated that both porphyrins in the hemocoel increased PO activity in the non-infected honeybees, suggesting activation of their immune system within 48 h after the porphyrin administration. However, the PO activity during this time was different for PP(Asp)_2_ and PP(Lys)_2_, i.e. the highest PO level was induced after 24 h by the former and after 48 h by the latter. This is probably associated with the greater ability of the porphyrin bearing aspartate moieties to penetrate into the hemocoel. The results of the AMP gene expression further reveal differences in the capability of the porphyrins to activate humoral immune response in honeybees (Fig. 7; Supplementary Table S3). It may have contributed to the differences in the spore reduction between the porphyrin treatments, which were noted especially on days 10 and 14 of the experiment (Fig. 2). Interestingly, a significant inverse correlation between the PO activity and the level of AMP gene expression was observed in the non-infected honeybees (and in the infected bees *versus* the infected control bees) within 24–48 h after the porphyrin administration (r =  − 0.61696; *p* = 0.0143). For each porphyrin, the highest PO activity was associated with a lower level of AMP gene transcripts and vice versa. Most likely, this corresponds to the mechanism targeted at achievement of a balance between the urgency to activate defense reactions and the feasibility of saving energy^52^.

To date, no work has been conducted on the impact of porphyrins on the immune response in invertebrates. The proPO system is known to be activated nonspecifically by small amounts of compounds of microbial origin, such as β-glucans, lipopolysaccharides, and peptidoglycans^10^. Induction of immune-related genes in response to other small molecules, e.g. arachidonic acid, benomyl, caffeine, sodium butyrate, herbicide pendimethalin, insecticide thiacloprid, fungicides fludioxonil and dimoxystrobin, and acaricide flumethrin, has been shown^33,53–57^. The dietary amino acid and vitamin complex (called “BEEWELL AminoPlus”) has been found to protect honeybees from immune suppression caused by *N. ceranae* by upregulating the expression of genes for abaecin, apidaecin, hymenoptaecin, defensin, and vitellogenin^12^. Silencing the naked cuticle (nkd) gene by double-stranded RNA specific to nkd in *N. ceranae*-infected bees activated the immune response, suppressed the reproduction of *N. ceranae*, and improved the health status of honeybees^58^.

In addition to direct inactivation of microsporidia, oral application of porphyrins in honeybees seems to stimulate host humoral immunity by activating PO. In turn, the cases of significant downregulation of the Aba and Def transcripts observed in the non-infected honeybees (Fig. 7a,b) may have been related to the elevated levels of PO activity and the immunomodulatory properties of porphyrins. The suppression of the bee immune system by the tested porphyrins should be excluded, because the healthy, non-infected honeybees exposed to these compounds showed a very similar survival rate to that in the porphyrin-untreated non-infected control bees (Fig. 3). Otherwise, the mortality of the honeybees would have increased significantly. In contrast to porphyrins, some natural extracts, organic acids, and bacteriocins exhibited high toxicity and were associated with increased mortality rates in laboratory cage tests^59,60^. A growing body of research suggests that various substances, including fungicides, can cause subtle yet significant harm to bees^61–63^.

Compared to healthy honeybees, *Nosema*-infected honeybees use a different tactic of immune response to porphyrin treatment. It is more complex because porphyrins directly inactivate microsporidian spores on the one hand and *N. ceranae* simultaneously influences PO activity and AMP gene expression in honeybees on the other hand. These differences were evident in the time of occurrence of the maximum PO activity in the honeybees fed with porphyrins (Fig. 6). The PO activity profile on days 3 and 4 p.i. in the infected bees was completely opposite to that in the healthy, non-infected bees (r =  − 0.5118, *p* = 0.000), which may be related to the changing load of *Nosema* spores. Notably, the increase in PO activity on day 4 p.i. (day 8 of the experiment) in the infected bees treated with PP(Asp)_2_ relative to the PP(Lys)_2_-treated bees (and the infected control bees) may have contributed to the faster rate of spore reduction by PP(Asp)_2_ over the next 2 days (day 10) compared to PP(Lys)_2_ (*p* < 0.01). The persistence of the higher PO level in the PP(Asp)_2_-treated bees may also partly explain the significantly lower expression of immune genes in this group of bees on day 4 p.i. than in the PP(Lys)_2_-treated honeybees (Fig. 7; Supplementary Table S3).

It should be emphasized that overexpression of immune genes is associated with an additional energy cost and can be damaging to bees. Excess of PO activity can induce several potentially dangerous highly reactive quinone intermediates^10^ whereby an inhibition system is then activated to control the immune self-stimulation thus avoiding possible excess of these metabolites^64^. This may lead to weakening of the insects, re-multiplication of *Nosema*, and in consequence increased bee mortality. It is worth noting that *N. ceranae* and neither of the porphyrins administered to the honeybees acted synergistically in the downregulation of the immune system in the infected honeybees, which could have caused harm to these insects. The survival of the infected honeybees was even significantly improved by the porphyrins (Fig. 3). In contrast, there is evidence that immunity and bee survival can be synergistically reduced by the combination of *Nosema* infection and some chemical exposure. For example, sublethal doses of fipronil, thiacloprid, and flupyradifurone insecticides highly increased the mortality of honeybees infected by *N. ceranae*^46,65^.

## Methods

The porphyrins were synthesized from protoporphyrin IX in accordance with the methodology described by Maximova et al.^66^. The concentration of the porphyrins applied in the experiments was determined on the basis of our previous investigations^42,44^.

1. *Course of the cage experiment with the honeybees*

Three combs with sealed brood were collected from one honeybee colony in the apiary at University of Life Sciences in Lublin. The combs were placed in an environmental chamber in the dark at 35 °C and humidified atmosphere (H = 60%). Emerging honeybees (*Apis mellifera carnica*) were collected and distributed in groups of 40 individuals into wooden cages (total 72 cages). One-day-old honeybees were checked for *Nosema* spores under light microscopy (Nikon ECLIPSE E200) and further confirmed to be free from *Nosema* by PCR, as described by Martín-Hernández et al.^67^, Chen et al.^21^, and Jack et al.^19^. The healthy honeybees were maintained for 3 days in laboratory conditions in darkness (25 °C; H = 65–70%). They were fed with a solution of sucrose (50% w/w in water) ad libitum until the infection by *N. ceranae* spores (days 3 and 4 of the experiment) (Fig. 1). Bees in half of the cages were left uninfected. The infection procedure was based on feeding the bees with sucrose-water syrup containing *N. ceranae* spores (10^7^ spores/mL) and lasted two days. The average quantity of spores administered to each bee was estimated from the total volume of the spore-containing syrup consumed by all bees in a cage (1.9 ± 0.13 mL per 2 days). The food was administered to the honeybees via syringes containing 5 mL of the sucrose solution.

The *N. ceranae* spores used for inoculation were isolated from intestines of experimentally infected honeybees following the method described by Sinpoo et al.^3^. The *N. ceranae*-infected honeybees were taken from selected colonies of the experimental apiary of the University of Life Sciences in Lublin^68^. The presence of *N. ceranae* DNA was demonstrated by detection of the specific 16S rDNA visualizing the amplified PCR products with a Taq PCR Core Kit (Qiagen)^19,67^ (Supplementary Fig. S1).

Two days post infection (p.i.), the honeybees assigned to the particular groups were given sucrose syrup containing porphyrins (100 µM) or pure sucrose syrup devoid of porphyrins. Six experimental groups (12 cages each) were created: (1) non-infected honeybees receiving no treatment (non-infected control bees), (2) non-infected bees treated with PP(Asp)_2_, (3) non-infected bees treated with PP(Lys)_2_, (4) honeybees infected with *N. ceranae* (infected control bees), (5) infected bees treated with PP(Asp)_2_, and (6) infected bees treated with PP(Lys)_2_.

The course of *Nosema* infection (expressed as spore loads per honeybee) was determined daily from day 6 to 8 of the experiment and then every second day (on days 10, 12, 14, and 16 of the experiment) according to Fries^69^ and Buczek et al.^43^. Briefly, ten live bees per experimental group were collected and pooled into one sample, which was homogenized in 10 mL of sterile distilled water. The spore number was counted using a hemocytometer chamber^24^. Every day, dead bees were counted and removed in order to assess survivability. The sucrose consumption was quantified and the feeders were replaced every second day.

Two independent cage experiments were performed (n = 480 bees per group; in total 2880 bees per one experiment).

2. *Preparation of biological material for the research*

Honeybees from two cages per group were sacrificed to isolate RNA and to collect hemolymph, respectively, for the analysis of the gene expression and PO activity 24 and 48 h after the administration of the porphyrins (i.e. days 3 and 4 post infection). Bees from one cage (about 35 specimens) were frozen in liquid nitrogen and stored at − 80 °C before use for determination of the expression of genes encoding antimicrobial peptides. The hemolymph was collected from honeybees from the second cage according to the method described by Borsuk et al.^70^, except that this work was carried out in a sterile cleanroom. Hemolymph was analyzed for the presence of porphyrin molecules and the level of PO activity.

A sample of 5 frozen bees and 5 bees from the second cage were examined by PCR to confirm the presence of *N. ceranae* spores.

3. *Measurements of the presence of porphyrin in intestines and hemolymph*

For visualization of porphyrin accumulation, the intestines from two infected and two non-infected live bees treated with each porphyrin were isolated at the end of the experiments. The intestines were placed on microscope slides and cut lengthwise with a scalpel under a light microscope to visualize the internal surface. The honeybee midgut tissue preparations were rinsed gently and extensively with sterile phosphate-buffered saline (PBS; in %: NaCl 0.8, Na_2_HPO_4_ 0.142, KCl 0.02, and KH_2_PO_4_ 0.027). The porphyrins in the honeybee intestines were identified by confocal microscopy (CLSM) using the laser system of the LSM780 Zeiss scanning confocal microscope. The microscopic analysis was carried out with a 405 nm laser for optimal excitation of the porphyrins and a PMT detector operating in the range of 600–700 nm corresponding to fluorescence emitted by porphyrins. In at least three different midgut regions emitting red fluorescence, emission spectra were collected using spectral imaging to confirm the presence of the porphyrin.

The presence of porphyrin in the hemolymph was determined by fluorescence spectroscopy. Hemolymph samples were maintained in darkness to prevent light activation until the spectral measurements. Fluorescence spectra of the hemolymph of the non-infected and *Nosema*-infected control honeybees fed with pure sucrose syrup and the honeybees fed with syrup containing porphyrin were registered. 3-µL aliquots of hemolymph were placed in a 2-mm quartz microcuvette and fluorescence signals of the porphyrins were registered at 23 °C using a Photon Technology International Inc. spectrofluorometer equipped with a continuous Xe-arc lamp as a light source and a photon counting detector. Fluorescence emission spectra were recorded in the range of 500–800 nm. The excitation wavelengths (λ_exc_ 404–406 nm) were from the range of the Soret band characteristic of PP(Asp)_2_ and PP(Lys)_2_. The spectral resolution of 1 nm was preserved and an integration time of 0.3 s was used.

4. *Immune-related gene expression*

The expression of immune-related genes encoding antimicrobial peptides, i.e. abaecin (Aba), defensin (Def), and hymenoptaecin (Hym), was measured in the honeybees on days 3 and 4 after infection (days 7 and 8 of the experiment). Two honeybees in each group were used for RNA extraction (performed at least three times for each experiment). Total RNA from dissected abdomens of honeybees was isolated using a GenElute Mammalian Total RNA Extraction Kit (Sigma), followed by DNase treatment (Turbo DNA-free, Life Technology). Reverse transcription was performed using 1 µg of total RNA with the use of random hexamer primers (High Capacity cDNA Reverse Transcription Kit, Life Technology). Quantitative RT PCR was performed using a Step One Plus PCR System (Applied Biosystems) and the Power SYBR Green PCR Master Mix (Applied Biosystems) according to the manufacturer’s instructions: 95 °C 10 min, 44 × (95 °C 15 s—denaturation, 60 °C 1 min—annealing and extension). Transcripts for actin, i.e. a housekeeping gene, were measured as a reference. The starters for actin, Aba, Hym, and Def were as follows:

**Table:** 

Gene/transcript	Forward: 5′–3′	Reverse: 5′–3′
Actin AB023025	GGAATGGAAGCTTGCGGTATT	TGCGATTCCAGGATACATGGT
Abaecin U15954.1	CGACAGTTGCATAAAACGGAAA	GACGTCCTGGTTGTGGTACATTT
Hymenoptaecin U15956.1	CGTTTCTGCTCAAGCGGAAT	TCCAAGGATGGACGACTTTTTC
Defensin NM_001011616.2	GACAGTGCTTGCGCTGCTAA	TAATGGCACTTAACCGAAACGTT

All products were of 129 bp length. As a standard curve, PCR amplification was performed with several dilutions of the DNA template from the infected control bees.

5. *Determination of phenoloxidase activity in hemolymph*

The phenoloxidase activity in the hemolymph of *A. mellifera* was determined on the basis of melanin formation as reported previously^51,71,72^. Briefly, 2 µl of twice diluted hemolymph without hemocytes was added to 18 µl of Tris-buffered saline (TBS; 50 mM Tris–HCl pH 6.8, 150 mM NaCl) with CaCl_2_. After 20-min incubation at room temperature, 180 µl of L-DOPA (2 mM L-dihydroxyphenylalanine in 50 mM PBS pH 6.5) was added and absorbance was measured at 490 nm over 90 min using a microtiter plate reader (Bio-Rad). The PO activity was determined in triplicate in three independent experiments.

6. *Statistics*

Statistical analysis was performed using TIBCO Statistica version 13.3 (TIBCO Software Inc, US). Comparisons between the levels of *Nosema* infection in the honeybees from the different experimental groups were performed using one-way ANOVA and Tukey’s post hoc test. Before the analysis, the normality of data distribution in each test group was examined using the Shapiro–Wilk test. The uniformity of the variance was checked using the Levene test. The PO activity and immune gene expression levels measured in the group of the infected bees 48 h after porphyrin administration were also tested by one-way ANOVA. The results of the levels of expression of the immune genes measured in the groups of healthy bees and infected bees 24 h after porphyrin administration did not meet the assumptions of the parametric test. Therefore, the results were analyzed using the ANOVA Kruskal–Wallis test, and significance between individual groups was assessed using comparisons of the mean ranks for all samples. Statistical significance was assumed at a *p* value of < 0.05. The mortality of the honeybees was analyzed by creation of Kaplan–Meier survival curves for the bees in each treatment. The curves were compared using a log-rank/Mantel–Cox post hoc test to determine which curves were significantly different from one another.

Spearman’s rank correlations were used for calculation of the correlations between the PO activity and the level of AMP gene expression.

## Conclusions

The present work confirms that *N. ceranae-*infected *A. mellifera* use systemic defense mechanisms against microsporidian infection consisting in activation of the proPO system and upregulation of some AMP genes during the early stage of parasite proliferation (up to 4 days post infection). It was shown that porphyrins penetrate into the hemolymph from the digestive system of the honeybees and have an impact on their immune system without adverse effects on the honeybee lifespan. The level of the humoral immune response depends on the health status of the bees and the level of *Nosema* infection. Porphyrins can stimulate the immune system of healthy insects by activation of humoral response. This implies that they may have great importance in the prevention of infections and *Nosema* reproduction^64,73^. In turn, in *N. ceranae*-infected honeybees, some porphyrin molecules are involved in direct action on the microsporidia in the midgut and others modulate immune defense responses in infected-honeybees creating optimal environmental conditions for elimination of pathogens (up to 4 days post infection). We suggest that, due to their immunomodulatory properties, porphyrins mobilize the honeybee immune system through induction of the optimal PO activity/AMP expression variant according to the varying level of nosemosis infection. Further activation of immune response in infected bees is prevented when the number of *Nosema* spores declines. Our findings indicate that the immune response to porphyrins contributes to a drastic reduction of *N. ceranae* spores in porphyrin-treated bees, allowing infected insects to improve their lifespan substantially. However, a further study including more factors (e.g. porphyrin concentrations and *N. ceranae* spore doses) is needed to determine the extent to which the elevated immune status of honeybees protects them from *Nosem*a infection and improves their fitness, considering the effects of the age of the honeybees. In order to obtain a better picture of the impact of porphyrins on bee wellness, further research will be focused on analyses of the porphyrin-treated honeybee gut microbiome, which serves important functions for honeybee health.

## Supplementary Information

Supplementary Information.

## Data Availability

The data generated or analyzed during this study are included in this article and its supplementary information files. Any further data that support the findings of this study are available from corresponding author on reasonable request.
