# Supplementary material for: Effect of amide protoporphyrin derivatives on immune response in Apis mellifera
Source: Sci Rep. 2022 Aug 24;12:14406. doi: 10.1038/s41598-022-18534-9 (PMC9402574; doi:10.1038/s41598-022-18534-9)
Supplement: Supplementary file 1 — Supplementary Information. [file 41598_2022_18534_MOESM1_ESM.docx]

**Effect of amide protoporphyrin derivatives** **on immune response in *Apis mellifera***

Mariusz Trytek^1^*, Katarzyna Buczek^1^, Agnieszka Zdybicka-Barabas^2^, Iwona Wojda^2^, Grzegorz Borsuk^3^, Małgorzata Cytryńska^2^, Agnieszka Lipke^4^, Dorota Gryko^5^

^1^Department of Industrial and Environmental Microbiology, Institute of Biological Sciences, Faculty of Biology and Biotechnology, Maria Curie-Skłodowska University, Akademicka 19, 20-033 Lublin, Poland

^2^Department of Immunobiology, Institute of Biological Sciences, Faculty of Biology and Biotechnology, Maria Curie-Skłodowska University, Akademicka 19, 20-033 Lublin, Poland

^3^Institute of Biological Basis of Animal Production; Faculty of Biology, Animal Sciences and Bioeconomy; University of Life Sciences in Lublin, Akademicka 13, 20-950 Lublin, Poland

^4^Department of Inorganic Chemistry, Institute of Chemical Sciences, Faculty of Chemistry, Maria Curie-Skłodowska University, M. Curie Skłodowska Sq. 2, 20-031 Lublin, Poland

^5^Institute of Organic Chemistry, Polish Academy of Sciences, Kasprzaka 44/52, 01-224 Warsaw, Poland;

*Corresponding author

E-mail 1: [mariusz.trytek@mail.umcs.pl](mailto:mariusz.trytek@mail.umcs.pl); e-mail 2: [mtrytek1@o2.pl](mailto:mtrytek1@o2.pl) (MT)

Department of Industrial and Environmental Microbiology, Institute of Biological Sciences, Maria Curie-Skłodowska University, 20-033 Lublin, Akademicka 19

Fax: +48-815375959; tel. +48-81-537-5933

**Supplementary Figure S1**

Experimental procedures. 2% agarose gels showing PCR products amplified from *Nosema* DNA extracted from spores isolated from intestines of *Nosema*-infected honeybees of selected colonies (C1-C6). M: Molecular weight markers (Gene ruler, 1-kb ladder, Thermo Scientific). Lanes C1-C5 correspond to selected colonies with *Nosema ceranae* infection. C6 represents a honeybee colony with *N. ceranae* and *N. apis* coinfection.

**
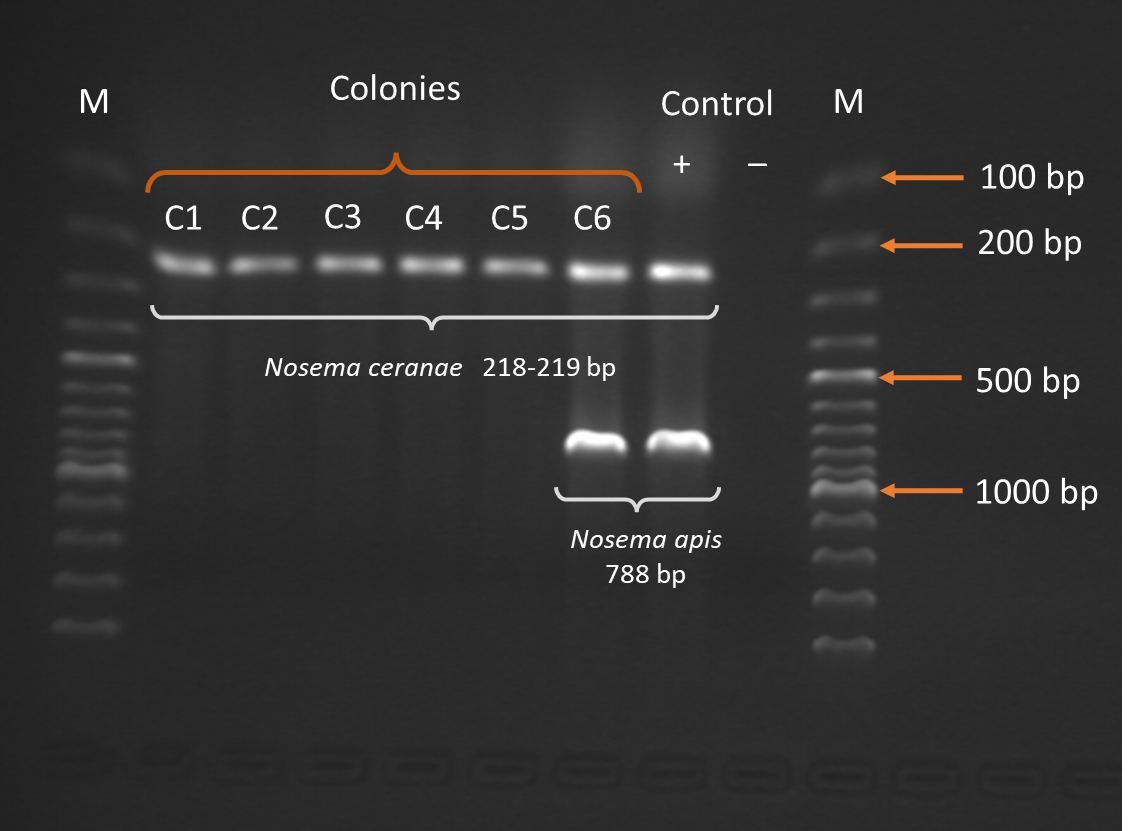
**

**Supplementary Figure S2**

Fluorescence spectra of the protoporphyrin IX amide derivatives, PP(Asp)2, and PP(Lys)2 in water.

**Supplementary Table S3**

P values of contrasts between the immune-related AMP gene expression in non-infected and infected honeybees treated with protoporphyrin IX derivatives on day 3 post *N. ceranae*-infection (data refers to Figure 7a).

|  |  |  | **Non- infected bees** | | | | | | **Infected bees** | | | | | | | | |
| --- | --- | --- | --- | --- | --- | --- | --- | --- | --- | --- | --- | --- | --- | --- | --- | --- | --- |
|  |  |  | PP(Asp)_2_ | | | PP(Lys)_2_ | | | Control | | | PP(Asp)_2_ | | | PP(Lys)_2_ | | |
|  |  |  | Aba | Def | Hym | Aba | Def | Hym | Aba | Def | Hym | Aba | Def | Hym | Aba | Def | Hym |
| **Infected bees** | PP(Lys)_2_ | Hym |  |  | p>0.05 |  |  | p>0.05 |  |  | p>0.05 |  |  | p>0.05 |  |  | - |
|  |  | Def |  | p<0.05 |  |  | p>0.05 |  |  | p>0.05 |  |  | p>0.05 |  |  | - |  |
|  |  | Aba | p<0.001 |  |  | p>0.05 |  |  | p<0.01 |  |  | p<0.05 |  |  | - |  |  |
|  | PP(Asp)_2_ | Hym |  |  | p>0.05 |  |  | p>0.05 |  |  | p>0.05 |  |  | - |  |  | p>0.05 |
|  |  | Def |  | p<0.05 |  |  | p>0.05 |  |  | p>0.05 |  |  | - |  |  | p>0.05 |  |
|  |  | Aba | p>0.05 |  |  | p>0.05 |  |  | p<0.001 |  |  | - |  |  | p<0.05 |  |  |
|  | Control | Hym |  |  | p<0.01 |  |  | p>0.05 |  |  | - |  |  | p>0.05 |  |  | p>0.05 |
|  |  | Def |  | p>0.05 |  |  | p>0.05 |  |  | - |  |  | p>0.05 |  |  | p>0.05 |  |
|  |  | Aba | p<0.0001 |  |  | p<0.0001 |  |  | - |  |  | p<0.001 |  |  | p<0.01 |  |  |
| **Non-infected bees** | PP(Lys)_2_ | Hym |  |  | p<0.01 |  |  | - |  |  | p>0.05 |  |  | p>0.05 |  |  | p>0.05 |
|  |  | Def |  | p<0.05 |  |  | - |  |  | p>0.05 |  |  | p<0.05 |  |  | p>0.05 |  |
|  |  | Aba | p<0.01 |  |  | - |  |  | p<0.0001 |  |  | p>0.05 |  |  | p>0.05 |  |  |
|  | PP(Asp)_2_ | Hym |  |  | - |  |  | p<0.01 |  |  | p<0.01 |  |  | p>0.05 |  |  | p>0.05 |
|  |  | Def |  | - |  |  | p<0.05 |  |  | p<0.05 |  |  | p<0.05 |  |  | p<0.05 |  |
|  |  | Aba | - |  |  | p<0.01 |  |  | p<0.0001 |  |  | p>0.05 |  |  | p<0.001 |  |  |

**Supplementary Table S4**

*P* values of contrasts between the immune-related AMP gene expression in non-infected and infected honeybees treated with protoporphyrin IX derivatives on day 4 post *N. ceranae*-infection (data refers to Figure 7b).

|  |  |  | **Non- infected bees** | | | | | | **Infected bees** | | | | | | | | |
| --- | --- | --- | --- | --- | --- | --- | --- | --- | --- | --- | --- | --- | --- | --- | --- | --- | --- |
|  |  |  | PP(Asp)_2_ | | | PP(Lys)_2_ | | | Control | | | PP(Asp)_2_ | | | PP(Lys)_2_ | | |
|  |  |  | Aba | Def | Hym | Aba | Def | Hym | Aba | Def | Hym | Aba | Def | Hym | Aba | Def | Hym |
| **Infected bees** | PP(Lys)_2_ | Hym |  |  | p<0.01 |  |  | p<0.01 |  |  | p>0.05 |  |  | p>0.05 |  |  | - |
|  |  | Def |  | p<0.01 |  |  | p<0.01 |  |  | p<0.05 |  |  | p>0.05 |  |  | - |  |
|  |  | Aba | p>0.05 |  |  | p>0.05 |  |  | p>0.05 |  |  | p>0.05 |  |  | - |  |  |
|  | PP(Asp)_2_ | Hym |  |  | p>0.05 |  |  | p>0.05 |  |  | p<0.001 |  |  | - |  |  | p>0.05 |
|  |  | Def |  | p>0.05 |  |  | p>0.05 |  |  | p<0.01 |  |  | - |  |  | p>0.05 |  |
|  |  | Aba | p>0.05 |  |  | p>0.05 |  |  | p<0.05 |  |  | - |  |  | p>0.05 |  |  |
|  | Control | Hym |  |  | p<0.001 |  |  | p<0.001 |  |  | - |  |  | p<0.001 |  |  | p>0.05 |
|  |  | Def |  | p<0.001 |  |  | p<0.001 |  |  | - |  |  | p<0.01 |  |  | p>0.05 |  |
|  |  | Aba | p<0.01 |  |  | p<0.01 |  |  | - |  |  | p<0.05 |  |  | p>0.05 |  |  |
| **Non-infected bees** | PP(Lys)_2_ | Hym |  |  | p>0.05 |  |  | - |  |  | p<0.001 |  |  | p>0.05 |  |  | p<0.01 |
|  |  | Def |  | p>0.05 |  |  | - |  |  | p<0.001 |  |  | p>0.05 |  |  | p<0.01 |  |
|  |  | Aba | p>0.05 |  |  | - |  |  | p<0.01 |  |  | p>0.05 |  |  | p>0.05 |  |  |
|  | PP(Asp)_2_ | Hym |  |  | - |  |  | p>0.05 |  |  | p<0.001 |  |  | p>0.05 |  |  | p<0.01 |
|  |  | Def |  | - |  |  | p>0.05 |  |  | p<0.001 |  |  | p>0.05 |  |  | p<0.01 |  |
|  |  | Aba | - |  |  | p>0.05 |  |  | p<0.01 |  |  | p>0.05 |  |  | p>0.05 |  |  |
